# Utilizing Wild *Cajanus platycarpus*, a Tertiary Genepool Species for Enriching Variability in the Primary Genepool for Pigeonpea Improvement

**DOI:** 10.3389/fpls.2020.01055

**Published:** 2020-07-23

**Authors:** Shivali Sharma, Pronob J. Paul, CV Sameer Kumar, Chetna Nimje

**Affiliations:** ^1^ Theme Pre-breeding, International Crops Research Institute for the Semi-Arid Tropics (ICRISAT), Hyderabad, India; ^2^ Regional Agricultural Research Station, Professor Jayashanker Telangana State Agricultural University, Palem, India; ^3^ Grain Quality Lab., International Crops Research Institute for the Semi-Arid Tropics (ICRISAT), Hyderabad, India

**Keywords:** *Cajanus platycarpus*, pre-breeding, introgression lines, wild *Cajanus* species, pigeonpea, grain nutrients, short-duration, photo-insensitivity

## Abstract

The use of crop wild relatives in the breeding program has been well recognized to diversify the genetic base along with introgression of useful traits. *Cajanus platycarpus* (Benth.) Maesen, an annual wild relative belonging to the tertiary genepool of pigeonpea, possesses many useful traits such as early maturity, high protein content, photoperiod insensitivity, and pod borer tolerance for the genetic improvement of cultivated pigeonpea. Using this cross incompatible wild *Cajanus* species, an advanced backcross population was developed following the embryo rescue technique. In the present study, a pre-breeding population consisting of 136 introgression lines (ILs) along with five popular varieties (used as checks) was evaluated for important agronomic traits during 2016 and 2017 rainy seasons and for grain nutrient content during 2016, 2017, and 2018 rainy seasons. Large genetic variation was observed for agronomic traits such as days to 50% flowering, number of pods per plant, pod weight per plant, grain yield per plant, and grain nutrients [protein content, grain iron (Fe), zinc (Zn), calcium (Ca), and magnesium (Mg)] in the pre-breeding population. Significant genotype × environment interaction was also observed for agronomic traits as well as grain nutrients indicating the sensitivity of these traits to the environments. No significant correlations were observed between grain yield and grain nutrients except grain Zn content which was negatively correlated with grain yield. Overall, 28 promising high-yielding ILs with high grain nutrient content were identified. These ILs, in particular, ICPP # 171012, 171004, 171102, 171087, 171006, and 171050 flowered significantly earlier than the popular mega variety, ICPL 87119 (Asha) and thus hold potential in developing new short-duration cultivars. The comprehensive multi-site assessment of these high-yielding, nutrient-rich accessions would be useful in identifying region-specific promising lines for direct release as cultivars. Moreover, these ILs are expected to replace the popular existing cultivars or for use as new and diverse sources of variations in hybridization programs for pigeonpea improvement.

## Introduction

Pigeonpea [*Cajajus cajan* (L.) Milllspaugh] is an important often-cross pollinated grain legume crop of semi-arid tropics grown under subsistence agriculture. Globally, it is cultivated on a 7.02 m ha area with an annual production of 6.81 m t (FAOSTAT, 2017) mainly in Asia, Africa, and Latin America. India is the largest producer and consumer of pigeonpea in the world where dry, dehulled split seeds are consumed as “daal”, a source of protein-rich (22–24%) food. Besides protein, pigeonpea seeds are also rich in carbohydrates, minerals, crude fibre, iron (Fe), sulphur, calcium (Ca), potassium (K), manganese (Mn) and water-soluble vitamins especially thiamine, riboflavin, and niacin (Saxena et al., 2010). In India, pigeonpea is the second most important legume after chickpea accounting for 5.39 m ha area and 4.87 m t of production (FAOSTAT, 2017). Besides mature seeds, immature green tender pods and seeds are consumed as vegetables mainly in Kenya, Tanzania, and Malawi. The crop is also grown for other uses such as fodder, medicine, rearing lac producing insects, fuelwood, and improving soil fertility through biological nitrogen fixation.

Narrow genetic base of cultivated pigeonpea and repeated use of a few elite breeding lines such as T-1 and T-90 (Kumar et al., 2004) in breeding programs are the major factors hindering its genetic improvement. Further, various biotic and abiotic stresses cause huge yield losses in pigeonpea worldwide and high levels of resistance/tolerance are not available in cultivated genepool. As a result, despite large breeding efforts in India and elsewhere, pigeonpea productivity is stagnant around 0.8-0.9 t ha^-1^. Major biotic stresses affecting pigeonpea are pod borers (*Helicoverpa armigera* Hubner, *Maruca vitrata* Geyer), and pod fly (*Melanagromyza chalcosoma* Spencer) among insect-pests and fusarium wilt (*Fusarium udum* Butler), sterility mosaic disease (SMD), and phytophthora blight (*Phytophthora drechsleri* Tucker) among diseases. Pigeonpea crop is also sensitive to abiotic stresses such as terminal drought, water-logging, salinity, and frost/cold. The protein advisory group of the United Nations has emphasized on improvement of the nutritional quality of proteins besides improving the productivity, adaptability, and yield stability of grain legumes.

One of the key factors for a successful crop improvement program is the availability of sufficient genetic variability. Over 13,200 accessions of cultivated pigeonpea and 555 accessions of wild species belonging to genus *Cajanus* from 60 countries are conserved in ICRISAT genebank. These germplasm accessions, based on the crossability relationship with cultivated pigeonpea, are grouped into three genepools with cultivated germplasm in the primary genepool (GP 1), all cross-compatible species, *C. acutifolius* (F.Muell.) Maesen*, C. albicans* (Wight & Arn.) Maesen*, C. cajanifolius* (Haines) Maesen*, C. cinereus* (F.Muell.)*, C. confertiflorus* F. Muell.*, C. lanceolatus* (W.Fitzg.) Maesen*, C. latisepalus* Maesen*, C. lineatus* (Wight & Arn.) Maesen*, C. reticulatus* (Dryand.) F.Muell.*, C. scarabaeoides* (L.) Thouars*, C. sericeus* (Baker) Maesen*, C. trinervius* (DC.) Maesen in the secondary genepool (GP 2), and the cross-incompatible species, *C. crassus* (King) Maesen*, C. goensis* Dalzell*, C. mollis* (Benth.) Maesen*, C. platycarpus* (Benth.) Maesen*, C. rugosus* (Wight & Arn.) Maesen*, C. heynei, C. kerstingii, C. volubilis, and other Cajaninae such as Rhynchosia* Lour., *Dunbaria W.* and *A., Eriosema (DC.)* Reichenb in the tertiary genepool (GP 3). Wild *Cajanus* species are the reservoirs of many useful genes/alleles and can be used to enrich variability in the primary genepool for developing new broad-based cultivars with increased plasticity (Sharma et al., 1993; Rao et al., 2003; Sharma and Upadhyaya, 2016; Sharma et al., 2019). Introgression of useful genes/alleles from the wild *Cajanus* species would help to break the yield plateau in pigeonpea. In chickpea, interspecific derivatives having high yield and resistance for wilt, foot rot, and root rot diseases (Singh et al., 2005) as well as for cyst nematode (Malhotra et al., 2002) were developed from crosses involving *C. reticulatum.* Similarly high‐yielding, cold‐tolerant lines with high biomass (ICARDA, 1995) and resistance to phytophthora root rot were developed from interspecific crosses involving *C. echinospermum* (Knights et al., 2008). Frequent utilization of wild *Cajanus* species in breeding programs is hindered due to cross incompatibility barriers and linkage drag. Among wild species, *C. platycarpus* is of particular interest to the pigeonpea breeders due to several useful traits such as extra‐early flowering and maturity (Saxena et al., 1996), photoperiod insensitivity, prolific flowering and podding, high harvest index, annuality and rapid seedling growth, and resistance/tolerance to biotic and abiotic stresses such as pod borer (Sujana et al., 2008), Fusarium wilt (Saxena et al., 1990), phytophthora blight (Ariyanayagam and Spence, 1978; Pundir and Singh, 1987; Dundas, 1990), nematodes (Sharma, 1995), sterility mosaic (Lava Kumar et al., 2005) and salinity (Subbarao, 1988). Using this cross incompatible wild *Cajanus* species, *C. platycarpus*, a backcross population was developed following embryo rescue technique (Mallikarjuna et al., 2011).

Linkage drag is the most common problem associated with the utilization of wild species in breeding programs. Hence, utilization of wild species in creating new genetic variability will be successful only when introgression lines (ILs) with useful traits and acceptable agronomic performance are developed and made available to breeders for direct use in breeding programs. Therefore, the present investigation was carried out a) to study the genetic variability in the advanced backcross population derived from *C. platycarpus* for important agronomic traits and grain nutrient, and b) to identify stable promising trait-specific ILs with minimum linkage drag for ready use in breeding programs to develop new cultivars with a broad genetic base.

## Materials and Methods

### Plant Material and Field Evaluation

Using a cross incompatible wild *Cajanus* species, *C. platycarpus* accession ICPW 68, and a popular pigeonpea cultivar ICPL 85010, a backcross population was developed following embryo rescue technique (Mallikarjuna et al., 2006). ICPW 68, originated from Uttar Pradesh, India, is extra-early flowering accession having high seed protein content (Saxena et al., 1996) and pod borer resistance (Sujana et al., 2008). ICPL 85010, also known as “Sarita”, is a short duration, determinate type pigeonpea variety having medium seed size (9.5 g 100-seed weight), which is cultivated in the Indian Subcontinent (Dahiya et al., 2001). Embryo rescue and tissue culture techniques were followed as described by Mallikarjuna and Moss (1995). The details of the population development have been documented by Mallikarjuna et al. (2006). The advanced backcross population consisting of 136 ILs in BC_4_F_10_ generation was used in this study. The 136 ILs along with five popular varieties (used as checks) of different maturity durations [ICPL 87119 (also known as “Asha”) ICP 8863 (Maruti), ICPL 20325, ICPL 85010, and ICPL 88039] were evaluated for different agronomic traits during the 2016 and 2017 rainy seasons and grain nutrients during the 2016, 2017, and 2018 rainy seasons at ICRISAT, Patancheru, Telangana, India (17°51′N, 78°27′E; 545 m). Among the checks, ICPL 87119 is a popular mega variety in the medium maturity duration group that is being widely cultivated in India over the past two decades; ICP 8863 is a medium-duration, high-yielding pigeonpea variety resistant to fusarium wilt which is popular in Karnataka, India; ICPL 20235 and ICPL 88039 are super-early and early maturing pigeonpea varieties, respectively. Accessions were planted in black soil (Vertisols) precision field in the first week of July in all years in an augmented design. Each check was placed after every 10 entries in each block and total lines were divided into three blocks. Each accession was sown in a single 4 m long row in a ridge and furrow system with a plant-to-plant spacing of 20 cm and row to row spacing of 75 cm. A standard package of practices was followed to raise a healthy crop. Manual weeding and spraying of insecticide were done to control weeds and insect-pests damage. The weather data of 2016 and 2017 crop season at ICRISAT, Patancheru, India is given in **Supplementary Figure 1**. Data were recorded on eight agronomic traits [days to first flowering, days to 50% flowering, plant height (cm), number of primary branches, number of secondary branches, number of pods per plant, pod weight per plant (g), and grain yield per plant (g)], and five grain nutrients [protein content (%), iron (Fe in mg kg^-1^), zinc (Zn in mg kg^-1^), calcium (Ca in g kg^-1^), and magnesium (Mg in g Kg^-1^) content]. Data on grain nutrients and two agronomic traits namely days to first flowering, and days to 50% flowering were recorded on a plot basis, whereas data on remaining agronomic traits (plant height, primary branches per plant, secondary branches per plant, pods per plant, pod weight per plant and grain yield per plant) were recorded on five randomly selected representative plants per plot following pigeonpea descriptors (IBPGR and ICRISAT, 1993). All the lines were harvested and threshed manually.

### Estimation of Grain Nutrient Content

For estimating the nutrient contents of Fe, Zn, Mg, Ca, and protein in grains, seeds of 136 ILs along with five checks were cleaned thoroughly and special care was taken during cleaning to prevent contamination of seeds with dust and metal particles. Seeds were washed with distilled water for a few seconds and dried in hot air at 40°C for 2 h to remove the dust and metal particles. Well-cleaned random seed samples were used for estimating grain protein, Fe, Zn, Ca, and Mg contents at the Charles Renard Analytical Laboratory, ICRISAT, Patancheru, India. The four dietary minerals- Fe, Zn, Ca, and Mg contents were assessed by nitric acid and hydrogen peroxide digestion accompanied by inductively coupled plasma optical emission spectrometry (ICP-OES) (Wheal et al., 2011). The sulfuric acid-selenium digestion method was adopted for the estimation of grain protein followed by the estimation of total nitrogen (N) in a SKALAR SAN^++^ SYSTEM autoanalyzer and the measurement of protein percentage as N percent × 6.25 conversion factor (Sahrawat et al., 2002).

### Statistical Analysis

Eight agronomic traits and five grain nutrients were analyzed separately for each rainy season and pooled over the two seasons for agronomic traits, and three seasons for grain nutrients using residual maximum likelihood (REML) in GenStat 15 (https://www.vsni.co.uk/) in mixed model approach considering genotypes as random effect and environment as fixed effect. The significance of environments was tested using Wald's statistic. Variance components due to genotype ($\sigma_{g}^{2}$) and genotype × environment ($\sigma_{g\times e}^{2}$) interaction and their standard errors (SE) were estimated. Best linear unbiased predictors (BLUPs) were obtained for agronomic traits and grain nutrients for each accession for individual environment as well as pooled over the environments. Based on BLUPs, the range, mean, variances and broad-sense heritability (H^2^) were estimated. Phenotypic correlations were estimated to determine trait associations in GenStat 15. Path analysis was performed to estimate the direct effect of the traits towards grain yield using R Version 3.5.3 (R Project for Statistical Computing, http://www.r-project.org/). To avoid the multicollinearity issues, two independent traits, days to 50% flowering and pod weight per plant were excluded while performing path analysis. Using the R package *cluster* (Patterson and Thompson, 1971), the Euclidean dissimilarity matrix was constructed using agronomic traits and grain nutrients and the accessions were clustered following Ward's method. Further, accessions with high grain yield and high grain Fe, Zn, Ca, Mg, and protein content were identified. Using the Euclidian distance matrix, the most diverse accession pairs were identified for potential use as parents in pigeonpea crossing programs.

## Results

### Variance Components and Trait Variability

The REML analysis showed significant variations among ILs $\left(\sigma_{g}^{2}\right)$ for all the eight agronomic traits in both 2016 and 2017 rainy seasons and all the five grain nutrients in 2016, 2017 and 2018 rainy seasons indicating the presence of significant variability among the ILs for these traits. Pooled analysis also showed significant genetic variance $\left(\sigma_{g}^{2}\right)$ and significant G×E interactions for all agronomic traits and grain nutrients (**Table 1**).

**Table 1:** Variance components due to genotypes $\left(\sigma_{g}^{2}\right)$, genotype × environment ($\sigma_{g\times e}^{2}$) interactions and their standard errors (SE) for agronomic traits and grain nutrients of *C. platycarpus* derived introgression lines evaluated during 2016, 2017, and 2018 rainy seasons at ICRISAT, Patancheru.

Traits	2016 rainy season		2017 rainy season		2018 rainy season		Pooled over seasons			
	$\sigma_{g}^{2}$	SE	$\sigma_{g}^{2}$	SE	$\sigma_{g}^{2}$	SE	$\sigma_{g}^{2}$	SE	$\sigma_{g\times e}^{2}$	SE
**Days to first flowering**	146.61^**^	19.460	228.28^**^	28.030	–	–	143.51^**^	20.388	41.51^**^	6.634
**Days to 50% flowering**	166.66^**^	21.150	210.85^**^	26.698	–	–	146.23^**^	20.620	38.42^**^	6.680
**Plant height (cm)**	353.05^**^	44.970	406.56^**^	51.870	–	–	340.57^**^	44.170	36.98^**^	8.860
**Primary branches (no.)**	21.67^**^	4.536	15.81^**^	3.072	–	–	8.37^**^	2.138	9.42^**^	2.649
**Secondary branches (no.)**	21.65^**^	3.290	8.728^*^	3.443	–	–	6.97^**^	1.779	7.14^**^	2.354
**Pods per plant (no.)**	5777.00^**^	1054.000	1909.30^**^	353.200	–	–	2499.10^**^	448.900	1282.70^**^	380.600
**Pod weight per plant (g)**	1005.20^**^	147.200	259.43^**^	47.100	–	–	322.70^**^	66.240	306.23^**^	53.780
**Grain yield per plant (g)**	434.12^**^	70.420	115.31^**^	20.540	–	–	150.59^**^	29.960	123.10^**^	25.090
**Protein (%)**	1.52^**^	0.281	1.53^**^	0.244	1.25^**^	0.361	0.43^**^	0.115	1.05^**^	0.150
**Fe (mg kg^-1^)**	19.11^**^	2.813	11.39^**^	2.669	20.77^**^	2.730	8.91^**^	1.536	8.73^**^	1.099
**Zn (mg kg^-1^)**	11.04^**^	1.841	11.53^**^	3.170	10.77^**^	2.051	7.84^**^	1.218	2.77^**^	1.080
**Ca (g kg^-1^)**	0.16^**^	0.024	0.18^**^	0.026	0.21^**^	0.027	0.13^**^	0.018	0.05^**^	0.008
**Mg (g kg^-1^)**	0.01^**^	0.005	0.02^**^	0.003	0.02^**^	0.004	0.01^**^	0.002	0.004^*^	0.002

*Significant at P ≤ 0.05; **Significant at P ≤ 0.01.

A large variation was observed amongst the ILs for all the agronomic traits and grain nutrients in each season as well as in the pooled analysis (**Table 2**). It is evident that some of the ILs performed better than the popular variety ICPL 87119 and the recurrent parent ICPL 85010 for these traits. A flowering window of around 60 days (76–141 days range) was observed for days to 50% flowering showing substantial population variability while popular variety ICPL 87119 took ~123 days to 50% flowering. None of the ILs flowered earlier than the recurrent parent ICPL 85010 (67 days to 50% flowering). Large variation was also noted amongst the ILs for plant height (above 100 cm: ~128–272 cm) when compared with the popular variety ICPL 87119 (on an average 222 cm tall) (**Table 2**). Similarly, number of pods per plant were much higher in the ILs (up to 386 pods in 2016 and 303 pods per plant in 2017) compared to ICPL 87119 (average 171 pods per plant) and the cultivated parent ICPL 85010 (average 126 pods per plant) in both seasons. Similar pattern was observed in 2016, 2017, and in pooled analysis for other traits such as grain yield per plant (up to 83 g in ILs compared to 48 g in ICPL 87119 and 25 g in ICPL 85010), protein content (up to 23% in ILs compared to 19% in ICPL 87119 and 21% in ICPL 85010), Fe (up to 43 mg kg^-1^ in ILs compared to 32 mg kg^-1^ in ICPL 87119 and 33 mg kg^-1^ in ICPL 85010), Zn (up to 42 mg kg^-1^ in ILs compared to 29 mg kg^-1^ in ICPL 87119 and 41 mg kg^-1^ in ICPL 85010), Ca (up to 3.1 g kg^-1^ in ILs compared to ~1.0 g kg^-1^ both in ICPL 87119 and ICPL 85010), and Mg (up to 1.6 g kg^-1^ in ILs compared to ~1.3 g kg^-1^ in ICPL 87119, and 1.1 g kg^-1^ in ICPL 85010) (**Table 2**).

**Table 2:** Summary statistics for various traits in pigeonpea pre-breeding population evaluated during 2016, 2017, and 2018 rainy seasons at ICRISAT, Patancheru.

Seasons		Days to first flowering	Days to 50% flowering	Plant height (cm)	Primary branches (no.)	Secondary branches (no.)	Pods per plant (no.)	Pod weight per plant (g)	Grain yield per plant (g)	Protein (%)	Fe (mg kg^-1^)	Zn (mg kg^-1^)	Ca (g kg^-1^)	Mg (g kg^-1^)
**2016 rainy season**	**Mean**	96 ± 1.05	104 ± 1.11	202.0 ± 1.62	13 ± 0.45	7 ± 0.42	206± 7.08	74.0 ± 2.82	49.8 ± 1.90	21.4 ± 0.12	32.8 ± 0.39	35.2 ± 0.30	1.2 ± 0.04	1.2± 0.01
	**Range**	72- 131	76 -140	127.9- 247.0	7 - 23	0 - 18	70- 386	17.9 – 172.1	14.0 - 109.3	18.6 - 23.8	23.8 - 43.9	26.0- 42.0	0.7 - 2.8	1.0- 1.6
	**Heritability (%)**	95.1	97.0	96.7	81.4	90.6	85.2	91.7	88.6	84.9	91.7	87.8	91.4	67.5
	**ICPL 85010**	64	68	96.0	11	2	131	39.4	27.3	19.5	31.1	37.9	0.9	1.0
	**ICPL 87119**	104	112	204.2	17	16	185	66.1	52.4	18.3	30.7	27.4	0.8	1.0
**2017 rainy season**	**Mean**	111± 1.29	119 ± 1.25	240.1 ± 1.74	21 ± 0.38	5 ± 0.33	143 ± 4.08	49.1 ± 1.50	33.9 ± 1.00	21.1 ± 0.11	39.0 ± 0.33	33.5 ± 0.35	1.6± 0.04	1.4 ± 0.01
	**Range**	72 - 135	84-141	155.5 - 272.3	12 - 30	2 - 12	60 - 303	16.8- 99.3	10.7- 66.2	18.2 - 23.8	31.8 - 45.4	27.6 - 40.3	0.9 - 3.5	1.1 - 1.7
	**Heritability (%)**	98.4	97.0	96.6	83.5	63	84.8	85.4	85.9	89.0	78.2	73.7	91.5	88.0
	**ICPL 85010**	59	66	126.1	15	4	123	37.8	22.9	22.9	35.3	42.5	1.3	1.2
	**ICPL 87119**	125	133	239.7	28	13	158	56.3	43.5	19.2	32.9	31.1	1.3	1.4
**2018 rainy season**	**Mean**	–	–	–	–	–	–	–	–	20.2 ± 0.12	35.2 ± 0.4	33.9 ± 0.31	1.6 ± 0.04	1.4 ± 0.01
	**Range**	–	–	–	–	–	–	–	–	18.2 - 22.6	26.2 - 46.4	26.3 - 42.4	0.9 - 3.3	1.1 - 1.7
	**Heritability (%)**	–	–	–	–	–	–	–	–	66.2	95.4	84.0	95	81.4
	**ICPL 85010**	–	–	–	–	–	–	–	–	20.1	34.1	40.1	1.1	1.2
	**ICPL 87119**	–	–	–	–	–	–	–	–	19.3	32.3	30.0	1.5	1.4
**Pooled over seasons**	**Mean**	103 ± 1.32	111 ± 1.25	221.1 ± 1.69	17 ± 0.5	6 ± 0.54	175 ± 5.10	61.6± 1.86	41.9 ± 1.28	20.9 ± 0.78	35.7 ± 0.04	34.2 ± 0.51	1.5 ± 0.04	1.3± 0.01
	**Range**	72 - 135	76 - 141	138.8 - 255.9	11 - 26	1 - 14	64- 321	18.8 - 123.3	13.1 - 82.9	19.3 - 23.0	26.4 - 42.5	27.6 - 41.8	0.9 - 3.1	1.1 - 1.6
	**Heritability (%)**	85.7	86.4	93	54.4	54.7	71.6	63.4	65.3	49.3	72.0	80.5	85.2	78.0
	**ICPL 85010**	61	67	110.3	13	3	126	38.2	24.7	20.8	33.4	40.6	1.1	1.1
	**ICPL 87119**	115	123	222.0	23	15	171	61.4	48.2	18.9	31.8	29.3	1.2	1.3

### Associations Between Agronomic Traits and Grain Nutrients

**Figure 1:**
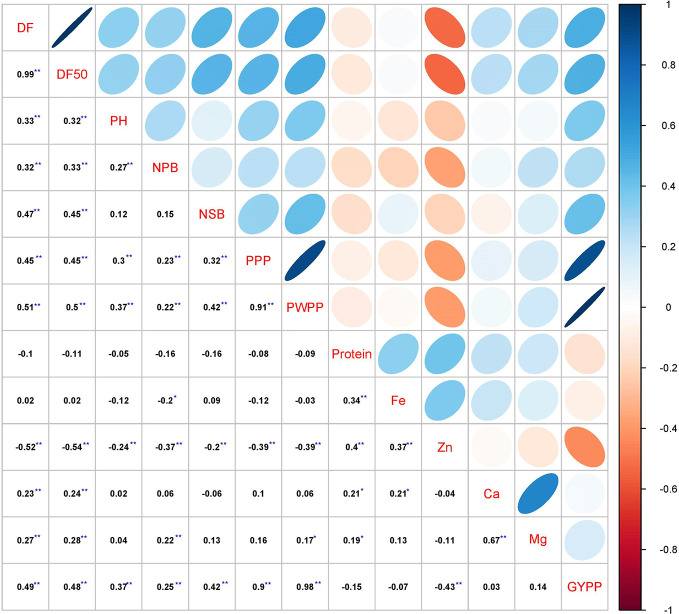
Correlation analysis of various agronomic and grain nutrient traits in pigeonpea pre-breeding population derived from *C. platycarpus* at ICRISAT, Patancheru. Days to flowering (DF & DF50), plant height (PH), primary branches (NPB), secondary branches (NSB), pods per plant (PPP), and pod weight per plant (PWPP) were positively associated with grain yield per plant (GYPP). Zn concentration was correlated negatively with grain yield per plant. On the other hand, no significant relationship was found for grain yield per plant with grain protein content, grain Fe, Ca and Mg content. It was observed that grain Fe and Zn as well as grain Ca and Mg were positively correlated. ^**^Significant at *P* ≤ 0.01; ^*^Significant at *P* ≤ 0.05.

The correlation analysis in 2016 (**Table S1**), 2017 (**Table S1**), and pooled analysis over years (**Figure 1**) showed that grain yield per plant was significantly and positively associated with days to first flowering (r = 0.38 in 2016, 0.42 in 2017 and 0.49 in pooled), days to 50% flowering (r = 0.40 in 2016, 0.39 in 2017 and 0.48 in pooled), plant height (r = 0.24 in 2016, 0.42 in 2017 and 0.37 in pooled), number of secondary branches (r = 0.50 in 2016, 0.24 in 2017 and 0.42 in pooled), pods per plant (r = 0.92 in 2016, 0.84 in 2017 and 0.90 in pooled), and pod weight per plant (r = 0.99 in 2016, 0.95 in 2017 and 0.98 in pooled) (**Table S1**, **Figure 1**). Number of primary branches had significantly positive correlation with grain yield per plant in 2017 (r = 0.45) and pooled over years (r = 0.25) but no correlation was observed in 2016 rainy season.

Similarly, among the grain nutrients, protein content in seeds showed significantly positive association with three nutrients, Fe (r = 0.23 in 2016, 0.32 in 2017, 0.34 in pooled), Zn (r = 0.44 in 2016, 0.32 in 2017, 0.40 in pooled), and Mg (r = 0.19 in 2016, 0.21 in 2017, 0.19 in pooled). Significantly positive correlation was observed between Fe and Zn content (r = 0.37 in 2016, 0.27 in 2017 and 0.37 in pooled) and between Ca and Mg content (r = 0.64 in 2016, 0.60 in 2017 and 0.67 in pooled) in year-wise and pooled analysis.

In the present study, no significant correlation was found between grain yield per plant with grain nutrients (Grain protein, Ca, Fe) in 2016 (**Table S1**), 2017 (**Table S1**), and pooled over years (**Figure 1**). Grain yield per plant showed a significantly negative correlation (r = -0.28 in 2016, -0.42 in 2017, and -0.43 in pooled) with Zn content. Further, the path analysis in 2016 (**Table S2**), 2017 (**Table S2**), and pooled analysis over the years revealed that pods per plant is the major direct contributor to grain yield (0.872 in 2016, 0.765 in 2017, and 0.813 in pooled). The other two traits which showed a major contribution towards yield were number of secondary branches per plant and plant height (**Table S2**).

### Identification of Promising Introgression Lines (ILs)

To understand the potential of these ILs derived from wild species in improving cultivated pigeonpea, the average performance of these lines over two years was compared with the popular variety, ICPL 87119 and the recurrent parent, ICPL 85010. None of the ILs flowered earlier than ICPL 85010 (days to 50% flowering <70 days). However, 104 ILs flowered significantly earlier (days to 50% flowering: 81–119 days) than ICPL 87119 (123 days). The majority of the ILs (> 100 lines out of 136 lines) were found significantly better than recurrent parent ICPL 85010 for most of the agronomic traits such as pods per plant, pod weight per plant and grain yield per plant (**Table 3**). In comparison with ICPL 87119 (~48 g), 28 ILs (~20% of the backcross population) had significantly higher grain yield per plant (~52–83 g) (**Table 3**). Besides, many ILs were found significantly better than ICPL 87119 in terms of plant height (50 ILs with 139–218 cm height), number of pods per plant (53 ILs with 189–321 pods) and pod weight per plant (55 ILs with 67–123 g). A few ILs also had a higher number of primary branches compared to ICPL 87119 (**Table 3**).

**Table 3:** Identification of promising trait-specific introgression lines derived from pigeonpea tertiary genepool species *C. platycarpus*.

Traits	Number of ILs significantly better than ICPL 87119 (range)	ICPL 87119	Number of ILs significantly better than ICPL 85010 (range)	ICPL 85010	Top five performing ILs against mega cultivar ICPL 87119 (range)
**Days to first flowering**	101 (74-110)	115	0	61	ICPP #171032, 171030, 171082, 171033, 171027 (74-77)
**Days to 50% flowering**	104 (81-118)	123	0	67	ICPP # 171082, 171032,171027, 171030, 171024 (81-85)
**Plant height (cm)**	50 (138.80-217.36)	221.99	0	110.32	ICPP # 171078, 171073, 171043, 171075, 171128 (138.80-193.62)
**Primary branches (no.)**	1 (26)	23	93 (16-26)	13	ICPP # 171022, 171037,171104, 171031, 171098 (24-26)
**Secondary branches (no.)**	0	15	63 (6-14)	3	ICPP # 171029, 171094, 171069, 171021, 171129 (12-14)
**Pods per plant (no.)**	53 (189-321)	171	101 (143-321)	126	ICPP # 171006, 171012, 171020, 171044, 171050 (288-321)
**Pod weight per plant (g)**	55 (66.57-123.30)	61.38	107 (43.64-123.30)	38.21	ICPP # 171119, 171012, 171006, 171088, 171020 (103.66-123.30)
**Grain yield per plant (g)**	28 (51.99-82.87)	48.18	111 (29.44-82.87)	24.72	ICPP # 171119, 171012, 171020, 171088, 171029 (68.19-82.87)
**Protein (%)**	136 (19.33-23.01)	18.88	54 (21.10-23.01)	20.79	ICPP # 171053, 171014, 171039, 171132, 171115 (22.51-23.01)
**Fe (mg kg^-1^)**	104 (33.60-42.50)	31.75	80 (35.23-42.50)	33.36	ICPP # 171078, 171077, 171009,171090, 171076 (41.07-42.50)
**Zn (mg kg^-1^)**	102 (32.12-41.76)	29.33	0	40.56	ICPP # 171027, 171082, 171032, 171132, 171023 (39.42-41.76)
**Ca (g kg^-1^)**	74 (1.35-3.09)	1.20	88 (1.26-3.09)	1.11	ICPP # 171010, 171045, 171127, 171104,171077 (2.28-3.09^)^
**Mg (g kg^-1^)**	45 (1.35-1.59)	1.27	115 (1.22-1.59)	1.14	ICPP # 171077, 171014, 171022, 171051, 171127 (1.53-1.59)

For grain nutrients, all the 136 ILs were found to have higher protein content (~19–23%) than the popular variety ICPL 87119 whereas, 104 ILs (~34–42 mg kg^-1)^ in grain Fe content, 102 ILs (~32–42 mg kg-^1^) in grain Zn content, 74 ILs (1.35–3.09 g kg^-1^) in grain Ca content, and 45 ILs (1.35–1.59 g kg^-1^) in grain Mg content were found significantly found better than ICPL 87119 (**Table 3**). Above 50 ILs were found promising than ICPL 85010 for most of the grain nutrient contents except Zn content (**Table 3**). Top five trait-specific ILs for each agronomic trait and grain nutrients are given in **Table 3**.

A total of 28 promising high-yielding ILs, which performed better than the popular variety, ICPL 87119, were identified (**Table 4**). Most of these high-yielding ILs exhibited higher amounts of five grain-nutrient contents. Remarkably, one line ICPP 171012 was found early (50% flowering: 109 days) having a high number of pods per plant (301), pod weight per plant (108 g), grain yield per plant (73 g), along with better grain nutrient contents such as high protein (21%), grain Fe (35mg kg^-1^), grain Zn (36.16 mg kg^-1^), grain Ca (1.39 g kg^-1^), and grain Mg (1.30 g kg^-1^) content compared to the best check ICPL 87119. Similarly, ILs ICPP # 171004, 171102, 171087, 171006, and 171050 were found most promising in terms of early flowering (109–116 days), high yielding (~62–65 g) and other agronomic traits along with higher grain nutrient contents than ICPL 87119 (**Table 4**).

**Table 4:** Performance of 28 promising high-yielding introgression lines for important agronomic traits and grain nutrients.

Geno	DF50^#^	PPP	PWPP	GYPP	Protein	Fe	Zn	Ca	Mg
**ICPP 171119**	131	285^*^	123.30^*^	82.87^*^	20.78^*^	29.82	30.35^†^	0.93	1.19
**ICPP 171012**	109^*^	301^*^	108.45^*^	73.36^*^	20.86^*^	35.36^*^	36.16^*^	1.39^*^	1.30^†^
**ICPP 171020**	119^†^	294^*^	103.66^*^	70.31^*^	19.60^*^	33.88^*^	31.17^†^	1.20^†^	1.30^†^
**ICPP 171088**	127	260^*^	103.98^*^	69.96^*^	19.80^*^	33.82^*^	27.61	1.20^†^	1.23
**ICPP 171029**	121^†^	207^*^	97.43^*^	68.19^*^	21.32^*^	38.41^*^	32.97^*^	1.29^†^	1.35^*^
**ICPP 171044**	130	292^*^	101.16^*^	66.02^*^	21.81^*^	36.05^*^	33.44^*^	1.98^*^	1.39^*^
**ICPP 171004**	111^*^	248^*^	94.39^*^	64.58^*^	20.09^*^	33.89^*^	31.53^†^	1.46^*^	1.29^†^
**ICPP 171102**	116^*^	271^*^	90.16^*^	63.19^*^	20.91^*^	33.97^*^	31.40^†^	1.25	1.42^*^
**ICPP 171129**	117^*^	194^*^	88.80^*^	63.11^*^	21.11^*^	34.57^*^	31.52^†^	1.08	1.23
**ICPP 171087**	110^*^	196^*^	86.07^*^	62.50^*^	20.27^*^	26.35	29.73^†^	2.06^*^	1.41^*^
**ICPP 171006**	109^*^	321^*^	107.96^*^	62.20^*^	21.31^*^	38.90^*^	36.09^*^	1.60^*^	1.41^*^
**ICPP 171050**	116^*^	288^*^	96.41^*^	61.91^*^	20.86^*^	36.28^*^	31.51^†^	1.90^*^	1.31^†^
**ICPP 171098**	124	206^*^	89.29^*^	61.57^*^	19.72^*^	38.45^*^	29.49^†^	1.27^†^	1.36^*^
**ICPP 171094**	115^*^	223^*^	89.36^*^	60.21^*^	21.35^*^	39.50^*^	36.60^*^	1.49^*^	1.33^†^
**ICPP 171021**	112^*^	202^*^	87.82^*^	59.12^*^	20.89^*^	32.01^†^	32.47^*^	1.43^*^	1.31^†^
**ICPP 171025**	118^*^	218^*^	84.29^*^	58.88^*^	21.63^*^	37.59^*^	36.71^*^	2.05^*^	1.45^*^
**ICPP 171001**	110^*^	215^*^	81.37^*^	58.82^*^	20.05^*^	36.93^*^	36.01^*^	1.01	1.37^*^
**ICPP 171085**	112^*^	199^*^	89.20^*^	58.70^*^	22.16^*^	38.21^*^	36.06^*^	1.52^*^	1.47^*^
**ICPP 171048**	114^*^	273^*^	89.13^*^	57.53^*^	21.10^*^	37.40^*^	34.41^*^	1.77^*^	1.23
**ICPP 171045**	129	278^*^	89.86^*^	57.31^*^	20.60^*^	34.81^*^	31.38^†^	2.61^*^	1.47^*^
**ICPP 171131**	109^*^	231^*^	75.77^*^	56.51^*^	20.95^*^	29.98	32.03^†^	1.05	1.07
**ICPP 171059**	112^*^	202^*^	84.29^*^	55.83^*^	20.79^*^	31.06	34.03^*^	1.02	1.32^†^
**ICPP 171097**	110^*^	195^*^	81.31^*^	55.77^*^	20.08^*^	37.13^*^	35.46^*^	1.27^†^	1.34^†^
**ICPP 171046**	120^†^	247^*^	77.58^*^	53.90^*^	21.19^*^	39.00^*^	32.92^*^	1.60^*^	1.19
**ICPP 171127**	117^*^	216^*^	77.53^*^	53.13^*^	21.51^*^	35.26^*^	30.09^†^	2.39^*^	1.53^*^
**ICPP 171113**	128	181^†^	81.11^*^	53.03^*^	20.21^*^	40.05^*^	33.77^*^	1.54^*^	1.31^†^
**ICPP 171003**	114^*^	213^*^	75.03^*^	52.54^*^	19.50^*^	35.92^*^	35.95^*^	1.23^†^	1.31^†^
**ICPP 171011**	117^*^	204^*^	76.95^*^	51.99^*^	20.77^*^	34.25^*^	33.71^*^	1.63^*^	1.42^*^
**ICPL 87119**	123	171	61.38	48.18	18.88	31.75	29.33	1.20	1.27

^#^DF50, Day to 50% flowering; PPP, Number of pods per plant; PWPP, pod weight per plant; GYPP, Grain yield per plant. *Significantly better than popular variety ICPL 87119 (Asha) at P≤ 0.05; ^†^Better than ICPL 87119 on per se basis.

### Cluster Analysis

**Figure 2:**
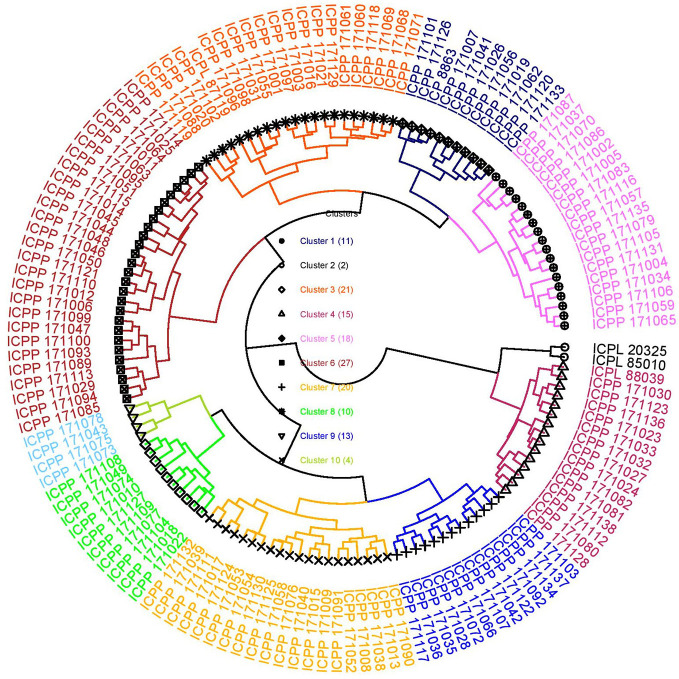
Cluster diagram depicting different clusters formed using 136 introgression lines derived from *C. platycarpus* and five popular varieties following Ward's method based on agronomic and grain nutrient traits. The “Cluster 2” consisted of only two lines: the recurrent parent ICPL 85010 along with another popular variety ICPL 20325. The highest yielding introgression line ICPP 171119 grouped in “Cluster 3” along with mega variety ICPL 87119.

Cluster analysis is performed to categorize lines into distinct groups/clusters wherein genotypes in different clusters are more diverse than within a cluster (Ward, 1963) and is useful in selecting the most diverse genotypes to be used as parents in crossing programs. In this study too, a hierarchical cluster analysis based on all eight agronomic traits and five grain nutrients over two seasons was performed to group the introgression lines into different clusters. The cluster analysis following Ward's method resulted in 10 clusters (**Table S3**; **Figure 2**). Cluster 6 was the largest cluster consisting of 27 ILs followed by cluster 3 (21 ILs) and cluster 7 (20 ILs). Cluster 2 had only two genotypes, ICPL 85010 and ICPL 20325; both are early maturing cultivars (50% flowering: 68 days) (**Figure 2**). All early maturing ILs were grouped into cluster 4 (50% flowering: 89 days). Cluster 3 had the highest cluster mean for grain yield per plant (55.12 g) followed by cluster 6 (53.33 g) whereas cluster 6 had the highest number of pods per plant (225) followed by cluster 3 (213). Cluster 10 exhibited the lowest means for grain yield per plant (~21 g) and pod weight per plant (~32 g) (**Table S3**). The popular mega variety, ICPL 87119 was grouped into cluster 3 along with the highest yielding introgression line ICPP I71119. Cluster 10 was found to be the best cluster in terms of high Fe content (40 mg Kg^-1^) but had the lowest grain yield per plant. Also, cluster 4 had highest mean for Zn content (38 mg Kg^-1^, “Cluster 2” has not been considered as it does not hold any ILs) and cluster 8 was found to have ILs with the maximum mean value for grain Ca (2.15 g Kg^-1^) and Mg (1.48 g Kg^-1^) content (**Table S3**).

The Euclidian distance matrix was estimated to identify the most diverse pair of ILs among the advanced backcross population as well as to identify the most similar and diverse ILs to the popular variety ICPL 87119 and the recurrent parent ICPL 85010 (**Table S4**). ICPP 171027 was found to be the most diverse (9.09) when compared with ICPL 87119, while ICPP 171031 (2.60) was the most similar IL with ICPL 87119. Likewise, ICPP 171119 was the most diverse (12.42) and ICPP 171033 was the most similar (5.68) accession to the recurrent parent ICPL 85010. Among the 136 ILs, the most diverse pair of accessions were ICPP 171119 and ICPP 171078 with a distance of 10.76. Top 10 most diverse pairs of accession are given in **Table S4**; ICPP 171119 was found to be the most diverse IL amongst these top 10 diverse pairs of ILs.

## Discussion

Grain legumes are an excellent and unique source of dietary protein for human beings in many parts of the world. The dietary importance of legumes is expected to increase over the years due to an increased demand for protein and other nutrients by the growing world population (Duranti, 2006). Moreover, the development of climate-resilient, nutrient-rich crop varieties is expected to reduce the number of malnourished people worldwide, especially in South Asia and Sub-Saharan Africa (Varshney et al., 2019). The crop varieties with improved nutrition may also be useful in addressing the UN Millennium goal of zero hunger and malnutrition, particularly in those parts of the world where plant-based protein is in high demand.

Pigeonpea is an excellent protein-rich legume crop that is mainly grown under rainfed conditions on marginal lands with minimal inputs and plays an important role in subsistence agriculture (Bohra et al., 2017; Obala et al., 2018). The modern pigeonpea cultivars have a narrow genetic base due to the frequent use of a few promising lines in the hybridization programs over the years. (Kumar et al., 2004; Bohra et al., 2010; Sharma et al., 2019). To meet the growing demand for plant-based nutrition and the need for soil health rejuvenation by implementing a proper cropping system, the pigeonpea breeding programs must embrace a few novel approaches (Anitha et al., 2019). The high-yielding nutrient-rich varieties will attract farmers not only in developing countries where the crop is being cultivated traditionally but will also find a niche in new environments in the developed countries (Atlin et al., 2017). Crop wild relatives (CWRs) are an excellent source of new alleles for different useful traits required for pigeonpea improvement (Khoury et al., 2015; Sharma and Upadhyaya, 2016; Sharma et al., 2019). But due to many hindrances such as cross incompatibility, late maturity, undesirable pod traits, poor agronomic performance, high photoperiod sensitivity, etc., breeders are disinclined to use these CWRs in crop improvement programs (Sharma et al., 2013). In this context, pre-breeding plays a vital role to enhance the use of wild relatives in breeding programs by providing the ready-to-use ILs with superior alleles for different traits introgressed from wild species.

The pre-breeding population consisting of 136 ILs used in the present study was derived from a cross-incompatible tertiary genepool species, *C. platycarpus* following embryo rescue technique (Mallikarjuna et al., 2011) with a view to introgress important traits such as early maturity, high protein content, and photoperiod insensitivity into cultivated pigeonpea. A large genetic variation was observed for important agronomic traits such as days to 50% flowering, number of pods per plant, grain yield as well as for the grain nutrients viz., protein, grain Fe, Zn, Ca, and Mg content. The genetic variation found in this backcross population is expected to be noble with broad genetic bases as it is derived from wild species.

Based on the days to maturity (duration from planting to 75% maturity), pigeonpea cultivars/varieties are grouped into different groups such as super-early (50–60 days to flowering and/or <100 days to maturity), extra-early (60–80 days to flowering and/or 101–120 days to maturity), early (81–100 days to flowering and/or 121–140 days to maturity), mid-early (101–120 days to flowering and/or 141–160 days to maturity), medium (111–130 days to flowering and/or 161–180 days to maturity) and late (> 130days to flowering and/or >180 days to maturity) maturity duration groups (Srivastava et al., 2012). In general, major pigeonpea cultivation is dominated by varieties in the medium-maturity group (Choudhary and Nadarajan, 2011). Under changing climatic conditions, there is an emphasis on developing short-duration pigeonpea cultivars having photo- and thermo-insensitivity to fit into multiple cropping systems as well as to expand pigeonpea cultivation into new niche areas (Saxena et al., 2019).

Though short duration lines are available, there is a huge yield penalty compared to popular medium-maturing varieties such as ICPL 87119. Most of the high-yielding ILs identified in the present study flowered early and had significantly higher yield and better grain nutrient contents than all the control cultivars used in this study including popular variety, ICPL 87119. The most promising high-yielding ILs such as ICPP # 171012, 171004, 171102, 171087, 171006, and 171050 having early flowering, high yield, and better grain nutrient contents hold great potential for ready use in pigeonpea breeding programs.

Dwarf plant type is not an advantageous feature for pigeonpea as it attracts *Helicoverpa armigera* and the dwarf bushy growth habit lines have shown 40% damage due to *H. armigera* (Mallikarjuna et al., 2011). Interestingly, all the ILs in this study were tall with semi-spreading secondary and tertiary branches and indeterminate growth habit. Plant height in pigeonpea is a complex and quantitative trait (Byth et al., 1981). The ILs evaluated were found taller than their recurrent parent ICPL 85010 and were similar to ICPL 87119 which puts the ILs in an advantageous position. Also, the range of yield-contributing traits such as number of pods per plant, pod weight per plant, and grain yield per plant was very high indicating the high level of recombination in these ILs.

Grain nutrients were found to be highly influenced by genotype and genotype × environment interactions. The stability of these traits in different environments is therefore important in crop improvement programs to improve the nutritional quality of pigeonpea. Non-significant correlations were observed between grain yield and grain nutrients except grain Zn content. This indicates the possibility to develop nutrient-rich pigeonpea varieties without the trade-offs for yield. Besides, a positive association was observed between the grain nutrients, such as of protein with all four grain nutrients suggesting the simultaneous improvement of varieties with enhanced multiple nutrient contents. Grain Fe and Zn content also showed a significant correlation which is reported in several crops including wheat (Morgounov et al., 2007), sorghum (Upadhyaya et al., 2016; Phuke et al., 2017), pearl millet (Kanatti et al., 2014), proso millet (Vetriventhan and Upadhyaya, 2018), and finger millet (Upadhyaya et al., 2011).

Further, it is important to study the similarity/dissimilarity of ILs with control cultivars for use in breeding programs. The cluster analysis grouped 136 ILs with 5 control cultivars into 10 clusters wherein similar ILs were placed in the same cluster based on agronomic traits and grain nutrients. This will help the breeders to choose trait-specific and diverse ILs for use in a breeding program to introduce new useful alleles derived from wild species into their working collection and/or newly developed cultivars/varieties. In addition, the most diverse pairs of ILs have been identified based on the mean phenotypic diversity index. Involving most diverse ILs in hybridization programs would be helpful in generating new and useful recombinants. Apart from this, a few ILs such as ICPP 171119, ICPP 171098, and ICPP 171045 showing maximum diversity with the recurrent parent ICPL 85010; and ICPP 171027, ICPP 171082, and ICPP 171024 having maximum diversity with the popular variety, ICPL 87119 have been identified for use in breeding programs to develop new cultivars with a broad genetic base.

## Conclusion

Significant variability was observed in the pre-breeding population, derived from the cross incompatible tertiary genepool species, *C. platycarpus,* for agronomic traits and grain nutrients. Moreover, it is noteworthy that many ILs performed better than the existing mega varieties not only in terms of yield but also for nutrient contents. The most promising high-yielding ILs such as ICPP # 171012, 171004, 171102, 171087, 171006, and 171050 having early flowering, high yield, and better grain nutrient contents compared to the best variety, ICPL 87119 hold great potential for ready use in pigeonpea breeding programs. A thorough multi-location evaluation of these promising trait-specific ILs will be efficacious in identifying region-specific promising lines for their possible direct release as a cultivar(s) having a diverse genetic base, especially in the short-duration group to fit into multiple cropping systems. Further, as no correlations were observed between grain yield and grain nutrients, it shows the possibility of developing nutrient-rich pigeonpea varieties without the trade-offs for yield. Positive associations of grain protein with all four grain nutrients suggests the simultaneous improvement of varieties with enhanced multiple nutrient contents. Besides this, the most diverse promising ILs can be included in the hybridization programs as the potential sources of new and diverse variations. Finally, as *C. platycarpus* is reported to possess photoperiod insensitivity, these ILs hold great potential for evaluation across locations and seasons to identify photo-insensitive lines for use in breeding programs.

## Data Availability Statement

All datasets generated for this study are included in the article/**Supplementary Material**.

## Author Contributions

SS conceived the idea and evaluated the material. CS was involved in field evaluation. PP analyzed the data and assisted in preparing the first draft. CN analyzed the seed samples for grain nutrient contents in quality lab. SS prepared the final manuscript. CS, PP, and CN provided their inputs. All authors contributed to the article and approved the submitted version.

## Funding

Funding support provided by the Global Crop Diversity Trust (GCDT) Grant Numbers GS15020 and GS18010 and CGIAR Research Program on Grain Legumes and Dryland Cereals (GLDC). 

## Conflict of Interest

The authors declare that the research was conducted in the absence of any commercial or financial relationships that could be construed as a potential conflict of interest.

The reviewer PK declared a past co-authorship with one of the authors SS to the handling editor.
