# Supplementary figures and images for: Utilizing Wild Cajanus platycarpus, a Tertiary Genepool Species for Enriching Variability in the Primary Genepool for Pigeonpea Improvement
Source: Front Plant Sci. 2020 Jul 23;11:1055. doi: 10.3389/fpls.2020.01055 (PMC7390956; doi:10.3389/fpls.2020.01055)

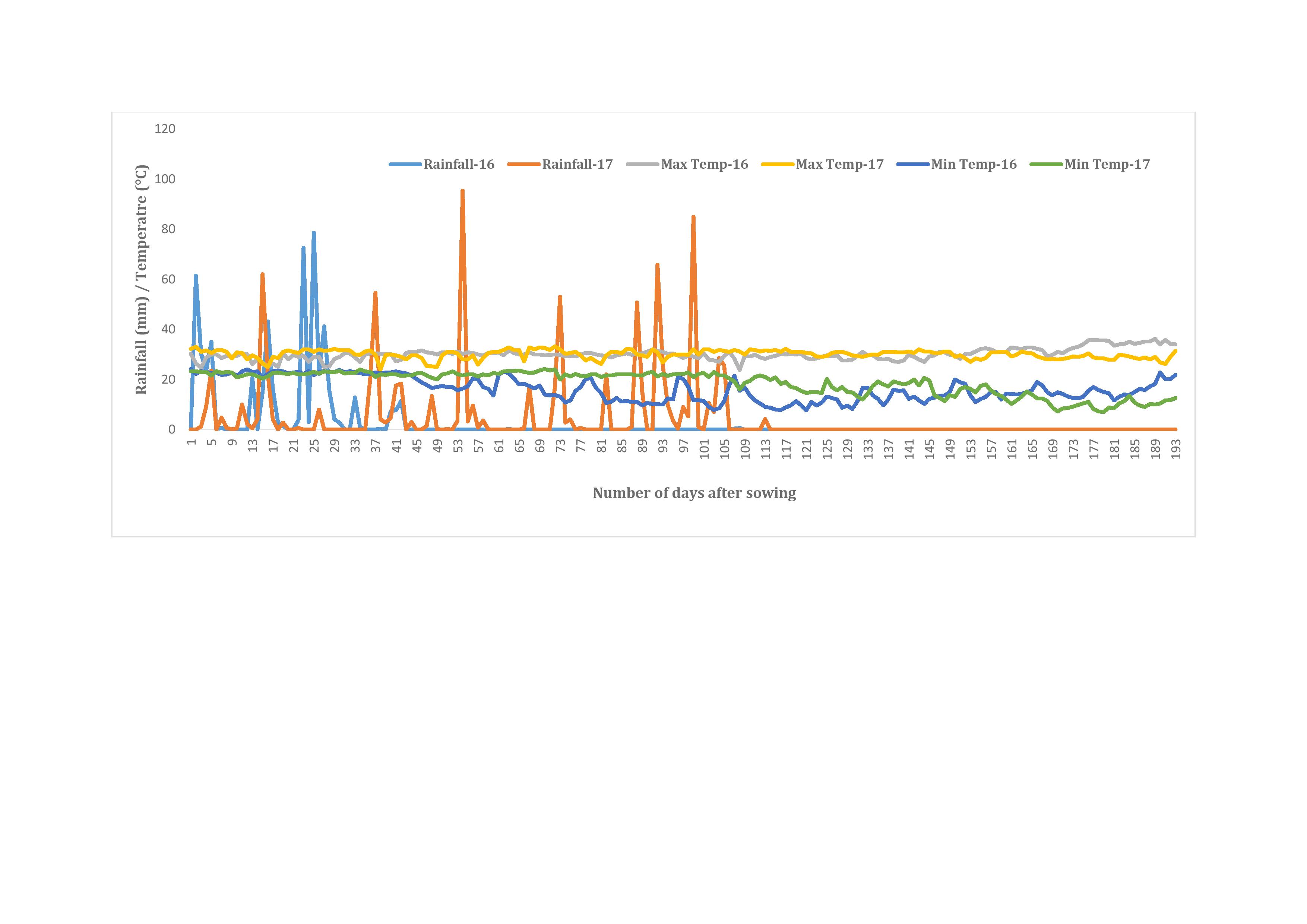

Supplement: Supplementary file 1 [file Image_1.jpeg]
